# Bibliometric and visual analyses of research on the links between stroke and exosomes from 2008 to 2023

**DOI:** 10.1097/MD.0000000000039498

**Published:** 2024-09-06

**Authors:** Jiaqi Song, Kaihan Zhan, Jiayu Li, Saiqi Cheng, Xiaohong Li, Li Yu

**Affiliations:** a School of Medical Technology and Information Engineering, Zhejiang Chinese Medical University, Hangzhou, China; b School of Pharmaceutical Sciences, Zhejiang Chinese Medical University, Hangzhou, China; c The Second School of Clinical Medicine, Zhejiang Chinese Medical University, Hangzhou, China; d The Fourth School of Clinical Medicine, Zhejiang Chinese Medical University, Hangzhou, China; e School of Basic Medical Sciences, Zhejiang Chinese Medical University, Hangzhou, China; f Key Laboratory of Drug Safety Evaluation and Research of Zhejiang Province, Hangzhou Medical College, Hangzhou, Zhejiang, China.

**Keywords:** bibliometric analysis, Chinese medicine, CiteSpace, cluster analysis, exosome, stroke

## Abstract

Exosomes, which are extracellular vesicles secreted and released from specific cells, exist widely in cell culture supernatants and various body fluids. This study aimed to analyze the research status of exosomes in stroke, and predict developmental trends via bibliometric analyses. The related literature from January 1, 2008 to January 1, 2024 was searched in the Web of Science Core Collection and 943 articles were retrieved. VOSviewer was used to visualize national cooperation and institutional cooperation. Cluster analysis of keywords and Citespace were applied for mutation analysis. Results: The analysis of 943 works of literature showed that the number of published articles has been steadily increasing since 2015. It is predicted that nearly 211 articles will be published in 2024 and 220 annually by 2028. China has the largest number of publications (473), followed by the United States (234), and Germany (61). The institution with the most publications is Henry Ford Hospital (Detroit, MI). In the keyword cluster “Exosomes and the Mechanism of Stroke: Inflammation and Apoptosis,” exosomes and inflammation were identified as hotspots. “Functional recovery” was a new trend in the keyword cluster of “Angiogenesis and Functional Recovery after Stroke.” China and the United States are the main forces in this field, and both countries focusing on drug treatments. The studies have been published mainly in China and United States. The findings of our bibliometric analyses of the literature may enable researchers to choose appropriate institutions, collaborators, and journals.

## 1. Introduction

Stroke includes ischemic stroke and hemorrhagic stroke^[1]^ and the high probability of disability and death is associated with. One quarter of strokes are recurrent.^[2]^ According to statistics, 1 person dies of a stroke every 3.5 minutes in the United States.^[3]^ Stroke is the leading cause of death and long-term disability in middle-aged and elderly people in China. In 2019, there were 3.94 million new cases of stroke in China, which directly resulted in 2.19 million deaths.^[4]^

Unfortunately, despite the very high incidence of stroke and other cerebrovascular diseases, there is still a lack of effective treatments. Currently available drugs are unable to repair the nerve damage caused by stroke.^[5–7]^ Presently, the treatment of ischemic stroke is limited to thrombectomy and the administration of tissue plasminogen activator (tPA),^[8]^ but these 2 methods have a narrow time window in which they are effective. Less than 10% of patients with stroke benefit from tPA treatment^[9]^ Intracerebral hemorrhage can be a complication of tPA administration, and it too is in urgent need of more effective treatment.^[10]^

The exosome is a type of biomarker and carrier of intercellular biological information.^[11]^ Exosomes can be used as carriers to deliver stroke drugs for repairing internal nerves and promoting angiogenesis.^[12]^ In addition, exosomes secreted by the mesenchymal stem cell (MSC) are related to inflammation regulation and neuroprotection. MSC-derived mixtures of exosome host proteins and RNAs have been identified, some of which are involved in anti-inflammatory, antigen presentation, or neuronal protection pathways.^[13,14]^ Furthermore, exosome microRNA plays a role in cerebral ischemia by promoting cell communication, regulating the nervous system, reshaping the blood vessels, and inhibiting neuroinflammation.^[15,16]^

Bibliometrics is a systematic and effective method for analyzing the scientific literature. Although this method has been applied to the subject of stroke, a bibliometric analysis that focuses specifically on the role of exosomes in stroke was needed to evaluate the current status of research in this field. In our study, bibliometrics was used to explore research directions and hotspots, as well as to predict publication trends. Similarities as well as differences in the number of publications, research directions, and cooperation between China and the United States were determined. This study aimed to elucidate the complete scenario in this field and provide a novel perspective for researchers.

## 2. Methods

### 2.1. Materials and methods

The data were obtained from the Web of Science (WoS), a database of scientific literature maintained by Clarivate Plc (London, UK). The database is a high-quality academic collection^[17]^ that provides its reference number, which is conducive to qualitative and quantitative analyses. The authors obtained the initial data from the WoS Core Collection (WoSCC). The language was set to English, and the literature type was selected as “article or review.” The search covered January 1, 2008 through January 1, 2024. All documents were retrieved on January 1, 2024 to avoid any bias due to the daily updates in the database.

The search strategy was as follows: Topic=(“stroke” or “intracerebral hemorrhage” or “ischemic stroke” or “brain infarction” or “brain stem infarction” or “cerebral infarction” or “cerebral stem infarction” or “ischemic encephalopathy” or “infarction encephalopathy” or “brain ischemia” or “brain stem ischemia” or “cerebral ischemia” or “cerebral stem ischemia”) AND Topic=(“exosome” or “exosomes” or “exocrine” or “extracellular vesicles ”or “derived exosome” or “external secrete body ”or “exocrine body” or “extracellular vesicle” or “plasma exocrine”).

### 2.2. Mathematical model and prediction

**Figure 1.**
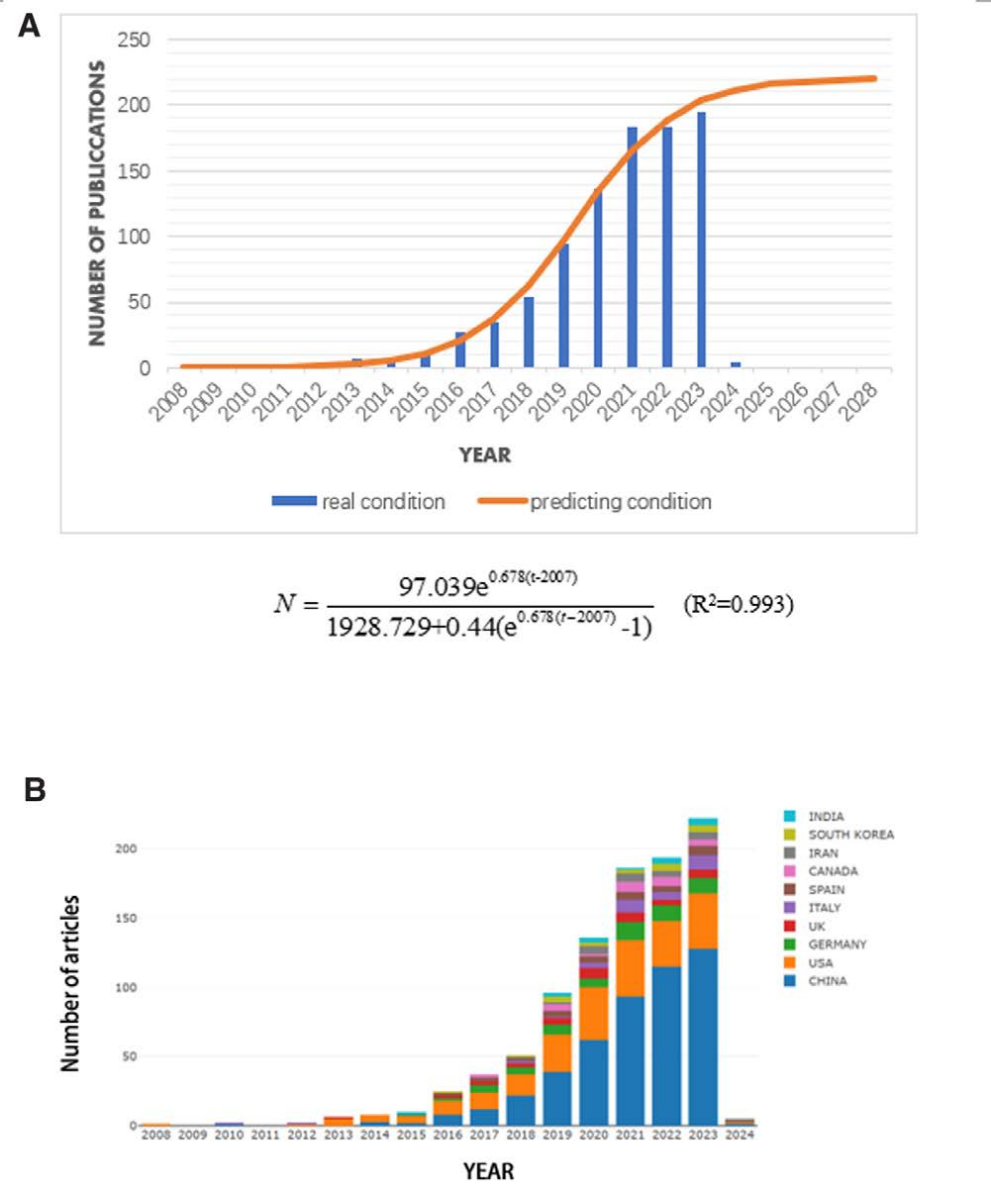
(A) Annual publication forecast curve (predicted condition) and the actual value (real condition).


Based on the data obtained for from 2008 to 2023, a mathematical model was used to perform nonlinear regression. As the annual publication volume was predicted, we estimated that it would increase every year at a certain rate, albeit it would not exceed a threshold. Therefore, we established a logistic growth model where N is the number of publications and t is the year of publication, according to the equation shown in Figure 1A.

$$\begin{array}{l} N=\frac{ae^{r\left(t-2007\right)}}{K+b\left(e^{r\left(t-2007\right)}-1\right)} \end{array}$$

### 2.3. Data analyses

The “Journal Citation Reports” (JCR) of the WoS were applied to evaluate the impact factor (IF) of journals. The “Essential Science Indicators” of the WoS was also used to evaluate the ranking of institutions. The data obtained from WoSCC were downloaded and imported into Excel, Bibliometric Online Analysis Platform (https://bibliometric.com), VOSviewer 1.6.18 (Centre for Science and Technology Studies, Leiden University, Leiden, Netherlands), and CiteSpace 6.2.R7 (https://citespace.podia.com/download). Excel was used to analyze the data of time and publication volume, and a model was established to predict the publication volume. Bibliometric Online Analysis Platform was applied to visualize the annual publication volume of different countries. VOSviewer was used to analyze the national and institutional cooperation and to visualize keywords. CiteSpace was applied to analyze the mutations of Chinese and American subject categories and the mutations of Chinese, American, and global keywords.

## 3. Results

### 3.1. Annual publications

Our search of WoSCC covered January 1, 2008 through January 1, 2024 and retrieved a total of 943 articles. From 2008 to 2012 (except 2009), 2 articles were published every year (Fig. 1A). From 2013 to 2023, the overall annual publication volume increased year-by-year. Furthermore, 2013 was probably a turning point because Hongqi Xin et al published their seminal article “Systemic Administration of Exosomes Released from Mesenchymal Stromal Cells Promote Functional Recovery and Neurovascular Plasticity after Stroke in Rats.” As of January 1, 2024, this article had been cited 678 times and has led the research in this field.

Therefore, the research field of exosomes in stroke has gradually become a hotspot. We have predicted and analyzed its annual publication volume. The obtained prediction curve and existing data as of January 1, 2024 are shown in Figure 1A. By fitting the established model, the relationship between time and annual publication volume was obtained. The goodness of fit (R²) was 0.993, which indicates a very high degree of fitting

According to our model, it is predicted that nearly 211 relevant articles will be published in 2024. Looking further ahead, it is expected that 220 papers will be published annually by 2028.

### 3.2. Publishing countries and regions

Articles about “exosome in stroke” have been published in 60 countries. Combined with the cited times, it can be seen that the United States contributed immensely to this research during 2012 to 2013, which led to the rise of this field. As of January 1, 2024, the top 3 countries for the total number of published articles were China (N = 473), the United States (N = 234), and Germany (N = 61).

We selected the top 2 countries, China and the United States, for further comparative analysis.

Since 2015, the annual number of publications in both countries has increased year-by-year (Fig. 1B). Interestingly, in terms of time span, the United States was the country to start the research earlier (2008) than China (2010) and contribute to the rise of the field. However, in terms of quantity, China has more publications than the United States (473 vs 234).

**Figure 2.**
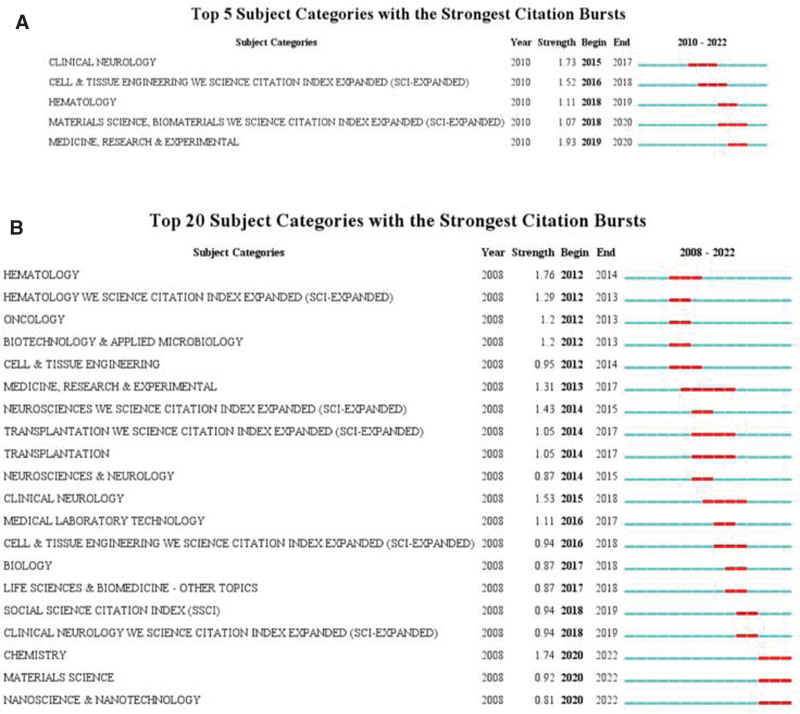
Subject categories with the strongest citation bursts. (A) Top 5 in China. (B) Top 20 in the United States.

From the perspective of research direction, in China and the United States, CiteSpace was used to analyze the mutation of the WoS discipline (subject) category. The top 5 subject categories with the strongest citation bursts in China are shown in Figure 2A. The top 20 subject categories with the strongest citation bursts in the United States are shown in Figure 2B. The latest and highest mutation intensity of the subject category in China is “Medicine, Research, & Experimental” during 2019 to 2020. The latest and very strong category mutations in the United States are “Chemistry,” and “Materials Science” and “Nanoscience & Nanotechnology” for the period 2020 to 2022. These findings illustrate the latest changes in the research directions of the 2 countries.

**Figure 3.**
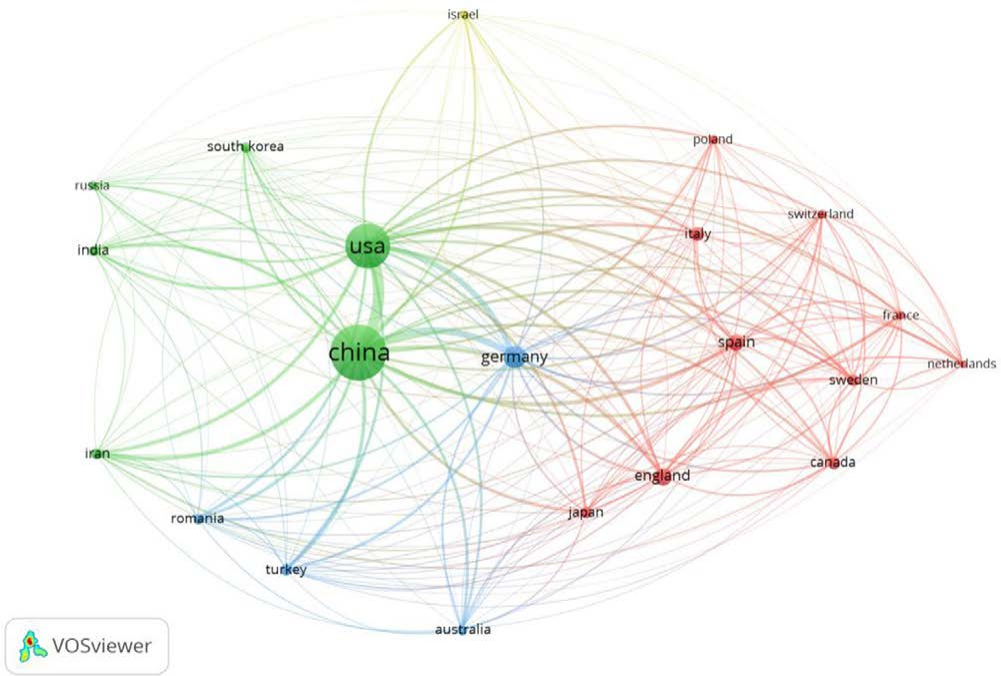
Analysis of national cooperation.

National cooperation in this research area was analyzed by VOSviewer (Fig. 3). China and the United States are the 2 most active countries in this field, and these 2 countries exhibit the highest cooperation (green circles). The total link strength of the United States is 65,039 and that of China is 73,376. Germany ranks third (yellow circle), with a total connection strength of 31,975. The link strength between China and the United States is 27,854, which is the closest cooperative relationship. Meanwhile, the intensity of cooperation between China and Germany ranks second (9355). In contrast, the connection strength of other countries is far less than this value, which is relatively not close.

From the perspective of national cooperation, it is not difficult to see that the close cooperative relationship between the United States and China (Fig. 3, thick green line) may be the reason for the similarities in their research directions. Both the total link strength and publication volume of Germany and the United Kingdom are lower than those of China and the United States, possibly because China and the United States are better at managing cooperative relations.

### 3.3. Citation frequencies

The citation frequency can reflect the influence of an article on a research field. The top 10 most-cited research papers (articles) about “exosomes in stroke” are shown in Table 1, with the top 8 considered by WoS to be highly cited. The top 10 most-cited research papers (reviews) are shown in Table 2. The most-cited article (678 citations as of January 1, 2024) is “Systemic administration of exosomes released from mesenchymal stromal cells promote functional recovery and neurovascular plasticity after stroke in rats.” It was published in 2013 by Hongqi Xin et al of Henry Ford Hospital (Detroit, MI). Among them, the latest achievement published in 2019 is that M2 microglia-derived exosomes protect the mouse brain from ischemia-reperfusion injury via exosomal miR-124, suggesting that exosomes derived from M2 microglia may be a new direction for the treatment of cerebral ischemia. The top 2 most-cited papers are Hongqi Xin articles published in 2012 to 2013 (Table 1), which signifies the author’s profound influence on this field. From a national perspective, it can be seen that the United States played an important role in this field in 2012 to 2013, thereby leading to its rise.

**Table 1:** Top 10 most-cited research papers (article).

Title	First author	Total citations	Journals	Institution	Publication year	Country	IF (2022)
Systemic administration of exosomes released from mesenchymal stromal cells promote functional recovery and neurovascular plasticity after stroke in rats	Hongqi Xin	678	Journal of Cerebral Blood Flow and Metabolism	Henry Ford Hospital	2013	USA	6.3
Exosome-mediated transfer of miR-133b from multipotent mesenchymal stromal cells to neural cells contributes to neurite outgrowth	Hongqi Xin	653	Stem Cells	Henry Ford Hospital	2012	USA	5.2
Surface functionalized exosomes as targeted drug delivery vehicles for cerebral ischemia therapy	Tian Tian	645	Biomaterials	Nanjing Medical University	2018	China	14
MiR-133b promotes neural plasticity and functional recovery after treatment of stroke with multipotent mesenchymal stromal cells in rats via transfer of exosome-enriched extracellular particles	Hongqi Xin	515	Stem Cells	Henry Ford Hospital	2013	USA	5.2
Extracellular vesicles improve poststroke neuroregeneration and prevent postischemic immunosuppression	Thorsten R Doeppner	514	Stem Cells Translational Medicine	University of Duisburg Essen	2015	Germany	6
Effect of exosomes derived from multipluripotent mesenchymal stromal cells on functional recovery and neurovascular plasticity in rats after traumatic brain injury	Yanlu Zhang	481	Journal of Neurosurgery	Henry Ford Hospital	2015	USA	4.1
Exosome mediated delivery of miR-124 promotes neurogenesis after ischemia	Jialei Yang	377	Molecular Therapy-nucleic Acids	Fourth Military Medical University	2017	China	8.8
MicroRNA cluster miR-17-92 cluster in exosomes enhance neuroplasticity and functional recovery after stroke in rats	Hongqi Xin	363	Stroke	Henry Ford Hospital	2017	USA	8.4
Elucidation of exosome migration across the blood-brain barrier model in vitro	Claire C Chen	310	Cellular and Molecular Bioengineering	University of California-Irvine	2016	USA	2.8
M2 microglia-derived exosomes protect the mouse brain from ischemia-reperfusion injury via exosomal miR-124	Yaying Song	257	Theranostics	Shanghai Jiao Tong University	2019	China	12.4

IF = impact factor.

**Table 2:** Top 10 most-cited research papers (review).

Title	First author	Total citations	Journals	Institution	Publication year	Country	IF (2022)
Circulating microRNAs: association with disease and potential use as biomarkers	Glen Reid	362	Critical Reviews in Oncology Hematology	The University of Sydney	2011	Australia	6.2
Exosomes-beyond stem cells for restorative therapy in stroke and neurological injury	Zheng gang Zhang	304	Nature Reviews Neurology	Henry Ford Hospital	2019	USA	38.1
Bidirectional microglia-neuron communication in health and disease	Zsuzsanna Szepesi	284	Frontiers in Cellular Neuroscience	Lund University	2018	Sweden	5.3
Molecular mechanisms responsible for therapeutic potential of mesenchymal stem cell-derived secretome	Randall Harrell	254	Cells	Regenerative Processing Plant LLC of Palm Harbor	2019	USA	6
Exosomes/miRNAs as mediating cell-based therapy of stroke	Hongqi Xin	228	Frontiers in Cellular Neuroscience	Henry Ford Hospital	2014	USA	5.3
Neutrophil extracellular traps villains and targets in arterial, venous, and cancer-associated thrombosis	Charlotte Thalin	220	Arteriosclerosis Thrombosis and Vascular Biology	Karolinska Institute	2019	Sweden	8.7
Neuroinflammation as a target for treatment of stroke using mesenchymal stem cells and extracellular vesicles	Sylwia Dabrowska	186	Journal of neuroinflammation	Warsaw University	2019	USA	9.3
Blood biomarkers for evaluation of perinatal encephalopathy	Ernest M. Graham	166	Frontiers in Pharmacology	Johns Hopkins University	2016	USA	5.6
The therapeutic potential of the mesenchymal stem cell secretome in ischemic stroke	Catriona J. Cunningham	155	Journal of cerebral blood flow and metabolism	University of Aberdeen	2018	England	6.3
Exosomes in stroke pathogenesis and therapy	Zheng Gang Zhang	152	Journal of Clinical Investigation	Henry Ford Hospital	2016	USA	15.9

IF = impact factor.

Co-cited references are considered to be a core component of bibliometric research. Co-citation is the frequency with which 2 documents are both cited together by another document.^[18]^ Co-cited references form the basis of articles in a field because research clusters begin to form when the same 2 papers are co-cited by many other papers. Table 3 lists the top 10 co-cited works of literature cited in this field, including their first authors and journals. The paper “Systemic Administration of Exosomes Released from Mesenchymal Stromal Cells Promote Functional Recovery and Neurovascular Plasticity after Stroke in Rats” (2013) is the most frequently co-cited paper, with a total of 217 times. The first author of this paper is Hongqi Xin. From Table 3, it is evident that Hongqi Xin as a first author appears repeatedly in the top 10 co-cited papers, appearing in a total of 4 papers (36.3%). This is not a common situation, which indicates that the author has extensive influence on the research basis in this field.

**Table 3:** Top 10 co-citation of cited references. Two references were tied for 8th place, each with 104 citations frequency.

Title	Country	Organization	Citation frequency	Journals	Publication year	First author
Systemic administration of exosomes released from mesenchymal stromal cells promote functional recovery and neurovascular plasticity after stroke in rats	USA	Henry Ford Hospital	217	Journal of Cerebral Blood Flow and Metabolism	2013	Hongqi Xin
Extracellular vesicles improve poststroke neuroregeneration and prevent postischemic immunosuppression	Germany	University of Duisburg Essen	160	Stem Cells Translational Medicine	2015	Thorsten R Doeppner
MicroRNA cluster miR-17-92 cluster in exosomes enhance neuroplasticity and functional recovery after stroke in rats	USA	Henry Ford Hospital	142	Stroke	2017	Hongqi Xin
MiR-133b promotes neural plasticity and functional recovery after treatment of stroke with multipotent mesenchymal stromal cells in rats via transfer of exosome-enriched extracellular particles	USA	Henry Ford Hospital	131	Stem Cells	2013	Hongqi Xin
Minimal information for studies of extracellular vesicles 2018 (MISEV2018): a position statement of the International Society for Extracellular Vesicles and update of the MISEV2014 guidelines	France	Universite PSL	130	Journal of Extracellular Vesicles	2018	Clotilde Thery
Exosome-mediated transfer of mRNAs and microRNAs is a novel mechanism of genetic exchange between cells.	Sweden	Department of Internal Medicine	125	Nature Cell Biology	2007	Hadi Valadi
Exosome-mediated transfer of miR-133b from multipotent mesenchymal stromal cells to neural cells contributes to neurite outgrowth	USA	Henry Ford Hospital	115	Stem Cells	2012	Hongqi Xin
Delivery of siRNA to the mouse brain by systemic injection of targeted exosomes	England	University of Oxford	104	Nature Biotechnology	2011	Lydia Alvarez-Erviti
Extracellular vesicles: exosomes, microvesicles, and friends	France	Institut Curie	104	Journal of Cell Biology	2013	Graça Raposo
Exosomes-beyond stem cells for restorative therapy in stroke and neurological injury	USA	Henry Ford Hospital	98	Nature Reviews Neurology	2019	Zheng gang Zhang
Surface functionalized exosomes as targeted drug delivery vehicles for cerebral ischemia therapy	China	Nanjing Medical University	97	Biomaterials	2018	Tian Tian

**Figure 4.**
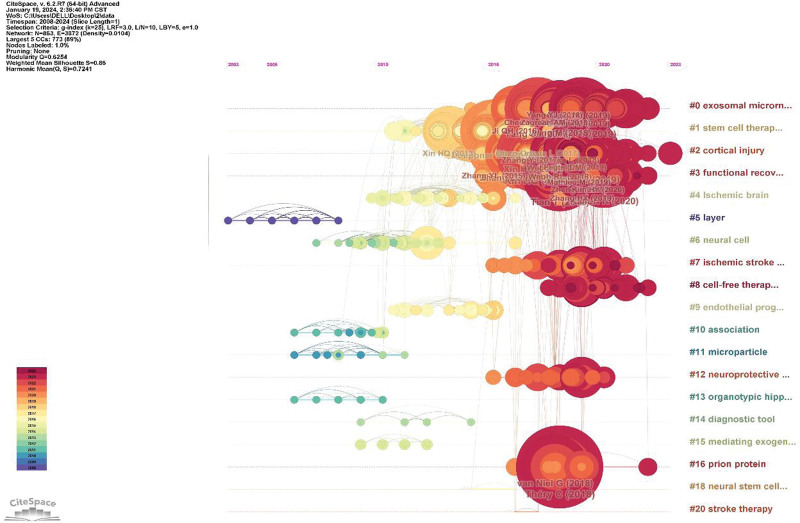
Timeline visualization of the reference co-citation map.

We used Citespace to analyze the co-cited references. The 943 articles retrieved from WoSCC were divided into 96 clusters. The largest 20 clusters are shown Figure 4, arranged vertically according to cluster size. The timeline is shown horizontally. The original cluster at the top of the figure is the latest cluster with high reference or reference mutation, but probably due to the wide range of research and cooperation in recent years, many clusters cannot extract features well. This reflects the consistency of research in this field from the side, perhaps indicating that there is no new mutation, but continues to dig into the original research. To gain a clearer insight into how they are grouped together, we included the tag content in Table S3, Supplemental Digital Content, http://links.lww.com/MD/N477. The clarity of keyword clustering results was judged by module value (Q-value) and average contour value (S-value). When Q > 0.3, the clustering structure is significant. When S > 0.5, the clustering result is reasonable; when ≥ 0.7, the clustering is highly efficient.^[19]^ The Q value of this cluster is 0.6254 and its S value is 0.86, which is clear and efficient.

### 3.4. Published journals

Articles examining the role of exosomes in stroke have been published in 372 journals. The top 10 active journals are listed in Table 4. From the total number of articles, it is clear that *International Journal of Molecular Sciences* is the largest contributor to the field, followed by *Stem Cell Research & Therapy*. The JCR partitions of these journals are in Q1 (58.8%) and Q2 (41.2%), which implies that this literature is innovative to a certain extent and that the published studies are relative leaders in the world. The IFs of these journals ranged from 3.4 to 10.8, with *Journal of controlled release* being the highest and *Frontiers in Neurology* the lowest.

**Table 4:** Top 10 journals with the largest number of publications.

Journals	Documents	Total citations	Citations per papers	Time span	Publishers	JCR partition (2022)	IF (2022)
*International Journal of Molecular Sciences*	41	484	11.80	2018.9–2023.12	MDPI	Q1	5.6
*Stem Cell Research & Therapy*	20	480	24	2019.11–2023.8	BioMed Central	Q2	7.5
*Frontiers in Cellular Neuroscience*	18	850	47.22	2014.11–2023.1	Frontiers Media S.A.	Q1	5.3
*Stroke*	17	981	57.70	2017.1–2023.12	Lippincott Williams and Wilkins Ltd.	Q1	8.4
*Translational Stroke Research*	16	761	47.56	2015.12–2023.12	Springer US	Q1	6.9
*Frontiers in Cell and Developmental Biology*	15	364	24.26	2020.7–2023.3	Frontiers Media S.A.	Q2	5.5
*Frontiers in Neurology*	15	447	29.8	2015.3–2023.9	Frontiers Media S.A.	Q2	3.4
*Neural Regeneration Research*	15	365	24.33	2017.1–2024.1	Wolters Kluwer Medknow Publications	Q2	6.1
*Molecular Neurobiology*	13	375	28.84	2016.4–2023.9	Springer	Q2	5.1
*Cells*	12	400	33.33	2019.5–2022.5	MDPI	Q2	6
*Experimental Neurology*	11	348	31.63	2018.11–2022.1	Academic Press Inc.	Q1	5.3
*Frontiers in Immunology*	11	142	12.90	2019.11–2023.1	Frontiers Media S.A.	Q1	7.3
*Frontiers in Neuroscience*	11	369	33.54	2018.6–2021.9	Frontiers Media S.A.	Q2	4.3
*Journal of Controlled Release*	11	272	24.72	2020.12–2023.11	ELSEVIER	Q1	10.8
*Journal of Nanobiotechnology*	11	295	26.81	2019.2–2023.11	BMC	Q1	10.2
*Biomedicines*	10	62	6.2	2020.8–2023.9	MDPI	Q2	4.7
*Journal of Cerebral Blood Flow and Metabolism*	10	1155	115.5	2013.11–2023.11	SAGE Publications Inc.	Q1	6.3
*Pharmaceutics*	10	140	14	2019.8–2023.12	MDPI	Q1	5.4

IF = impact factor, JCR = Journal Citation Reports.

### 3.5. Institutional cooperation

A total of 1267 institutions have published articles in this field. Henry Ford Health System was the most prolific institution, with 49 publications, followed by Henry Ford Hospital, one of its constituent units, with 48. Oakland University (Rochester, MI; <30 miles from Henry Ford Hospital) is third with 39 publications. The top 10 organizations with the highest number of publications are shown in Table 5. There are 5 organizations in the United States, 6 in China, and 2 in Germany.

**Table 5:** Top 10 most-productive organizations.

Organizations	Documents	Rank (ESI) (Neuroscience & Behavior)	Country
Henry Ford Health System	49	493	USA
Henry Ford Hospital	48	519	USA
Oakland University	39	956	USA
Shanghai Jiao Tong University	36	191	China
University of Duisburg Essen	25	262	Germany
Fudan University	23	179	China
Harvard University	23	2	USA
University of Gottingen	23	118	Germany
Southern Medical University China	22	404	China
Central South University	21	302	China
Capital Medical University	19	111	China
Southeast University China	17	657	China
University System of OHIO	17	42	USA

Three organizations were tied for 6th place, each with 23 articles. Two organizations were tied for 10th place, each with 17 articles. The institute’s Essential Science Indicators (ESI) ranking is the ranking of the field of Neuroscience & Behavior.

ESI = Essential Science Indicators.

**Figure 5.**
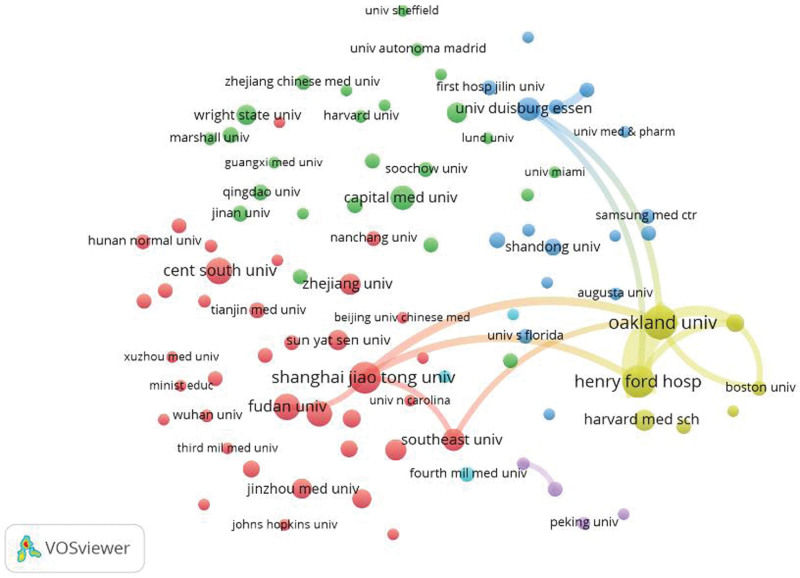
Organizational cooperation network.

We used VOSviewer to visualize the cooperation between institutions (Fig. 5). While 92 institutions are above the threshold of the minimum number of published articles (N = 5), and they exhibit cooperation. There are 1129 institutions that are below the threshold of 5 published articles, and they exhibit less cooperation with other organizations.

Moreover, the link strength of most institutions is not very high, implying that cooperation between institutions is concentrated in certain institutions (Table S1, Supplemental Digital Content, http://links.lww.com/MD/N477). The total link strength of Henry Ford Hospital is second (35,990), despite having with the highest number of articles (N = 49), and that of Oakland University is first (39,873), but with the third-highest number of posts (N = 39).

### 3.6. Co-occurrence of keywords

**Figure 6.**
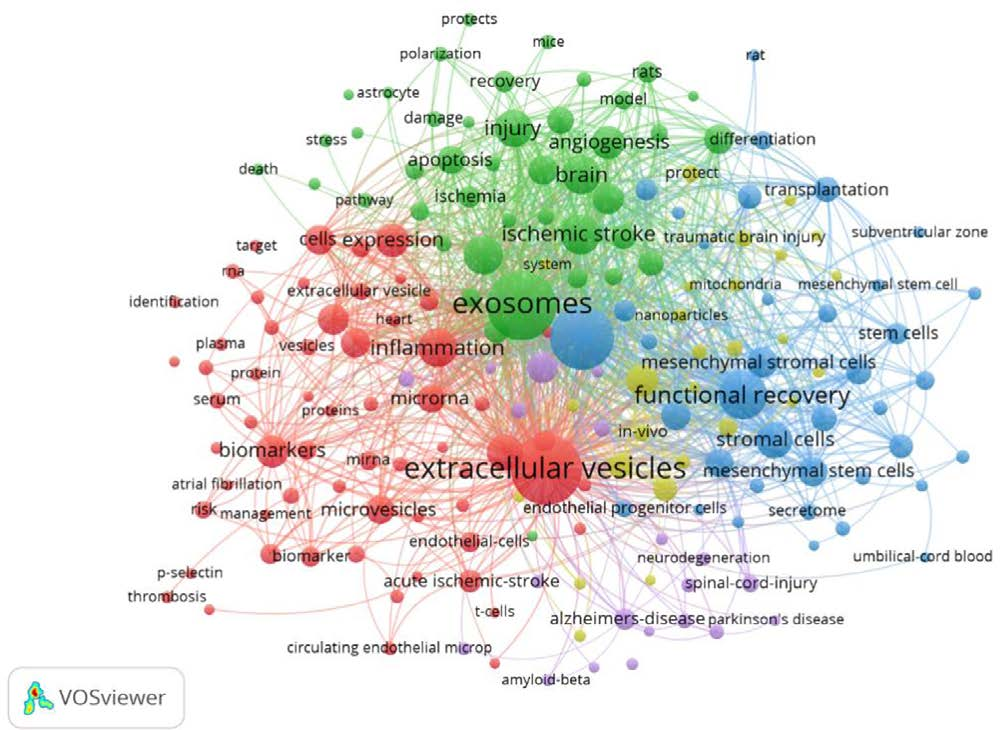
Clustering of keywords.

According to established search principles, keywords were selected that reflect the overall focus of the research.^[20]^ VOSviewer was used for keyword analysis (Fig. 6). Keywords were divided into 5 clusters: red, green, blue, yellow, and purple. The red cluster named “Exosomes and the Mechanism of Stroke: Inflammation and Apoptosis.” was the largest keyword cluster and has the main keywords of extracellular vesicles, inflammation, microrna, and biomarkers. The yellow cluster was titled “Brain Injury and Neurorepair,” with main keywords of exosomes, brain, ischemic stroke, and neurogenesis. The blue cluster was named “Angiogenesis and Functional Recovery after Stroke,” with main keywords of stroke, functional recovery, stem cells, and transplantation. The yellow cluster was titled “Treatment Strategies for Stroke,” and its main keywords are cerebral-ischemia, blood–brain-barrier, and in vitro. The purple cluster was titled “Associated nerve damage,” and its main keywords are neuroinflammation, alzheimers-disease, and spinal-cord-injury.

The criterion of at least 10 occurrences of all keywords was chosen, and 190 keywords met this criterion. The top 5 keywords were “extracellular vesicles,” “exosomes,” “stroke,” “functional recovery,” and “ischemic stroke.” It is worth noting that, aside from the topic-related keywords, “stromal cells” ranks 8th (Table S2, Supplemental Digital Content, http://links.lww.com/MD/N477). Taken together, these results should help researchers to check the hot spots in the field of stroke, and turn to the research of stroke recovery, exosome treatment and stem cell therapy.

**Figure 7.**
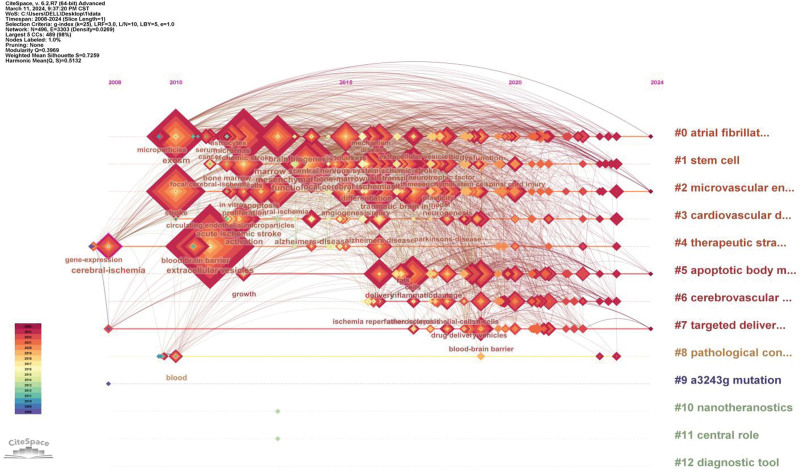
Timeline visualization of the keyword co-citation map.

CiteSpace was used for the timeline analysis of keywords. Keywords were divided into 12 clusters (Fig. 7). To gain a clearer insight into how they are grouped together, we included the tag content in Table S4, Supplemental Digital Content, http://links.lww.com/MD/N477. These clusters were different from those in the VOSviewer analysis to some extent, but we could see that there were similarities for the keywords of stem cells and extracellular vesicles. The top keyword cluster was extracellular vesicle. The value of Q is 0.3969 and the value of S is 0.7259, which indicates that this cluster is relatively stable. From the timeline analysis, brain–heart interaction, mesenchymal stem cells, blood–brain barrier integrity and stem cell therapy are all relatively new keyword clusters, while blood–brain barrier integrity is a cluster with a longer duration.

**Figure 8.**
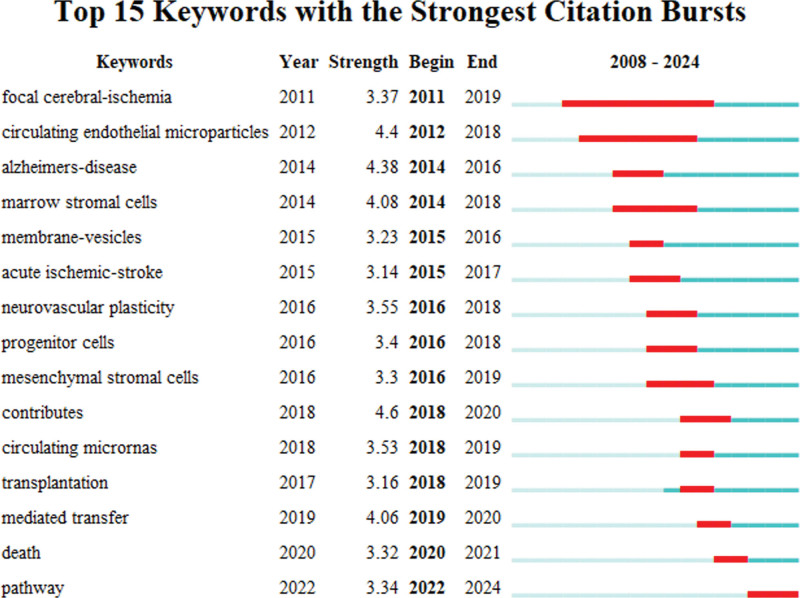
The top 15 keywords with the strongest citation bursts.

Moreover, the mutation of keywords can reflect the sudden change in the citation frequency of keywords at a certain time and lock the frontier of this field for us. CiteSpace was employed to analyze the keyword mutations. Because the number of published articles is relatively small, the mutation intensity is small. The top 15 keywords with the strongest citation bursts are shown in Figure 8. The highest mutation intensity was 4.38 for Alzheimer disease and the time range of mutation was from 2014 to 2016. The most recent mutation, pathway, had an intensity of 3.34, with time range of mutation from 2022 to 2024.

**Figure 9.**
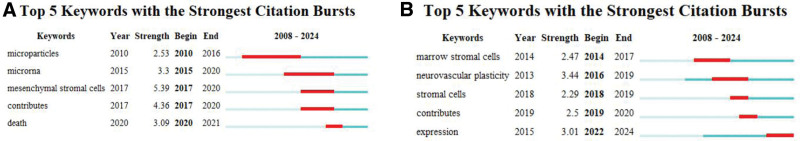
(A) Chinese keyword mutations. (B) American keyword mutations.

From the analysis of keywords, “stromal cells” appeared most frequently in both China and the United States. Using CiteSpace, the mutations of Chinese and American keywords were obtained using the same method. The keyword with the highest mutation intensity in China was “mesenchymal stromal cell” (strength = 5.39), with the mutation period of 2017 to 2020 (Fig. 9A). The keyword with the highest mutation intensity in the United States was “neurovascular plasticity” (strength = 3.44), with the mutation period of 2016 to 2019 (Fig. 9B).

Authorship There were a total of 4850 authors who had published articles about exosomes in stroke. The top 10 authors with the highest number of publications are listed in Table 6. Michael Chopp of the United States has the highest number of publications (N = 39) and citations (N = 4738). Thorsten R Doeppner of the Germany ranks second, with 21 articles and 1156 citations. Guoyuan Yang, Zhijun Zhang and Yaohui Tang are emerging authors in this field, as their average publication year is 2021 and beyond, and their numbers of publications are ranked in the top 10. Most of the top 10 prolific authors are based in the United States (N = 3) or Germany (N = 4), with 3 authors from China. Given that authors in China publish the largest total number of articles in the world, a possible reason there is only 3 authors in China in the top 10 prolific authors is because there are more authors in the country. This finding indicates that research in this field in China has become a hotspot, but it needs to be further deepened.

**Table 6:** Top 10 active authors with the most numbers of documents.

Authors	Publications	Citations	Average publication year	Average citations	Institutions	Countries
Michael Chopp	39	4738	2018.744	121.4872	Henry Ford Hospital	USA
Thorsten R Doeppner	21	1156	2020.524	55.0476	Istanbul Medipol University	Germany
Zheng Gang Zhang	21	3501	2018.333	166.7143	Henry Ford Hospital	USA
Dirk M Hermann	20	1235	2020.25	61.75	Univ Med Essen	Germany
GuoYuan Yang	16	643	2021.188	40.1875	Shanghai Jiao Tong University	China
ZhiJun Zhang	14	609	2021.071	43.5	Nantong university	China
Mathias Bähr	12	258	2020.667	21.5	University of Groningen	Germany
Bernd Giebel	12	985	2019.667	82.0833	University of Duisburg Essen	Germany
Yaohui Tang	11	414	2022	37.6364	Shanghai Jiao Tong University	China
Zhang Yi	11	1259	2019.636	114.4545	Henry Ford Hospital	USA

## 4. Discussion

This study focuses on the field of “role of exosomes in stroke” using bibliometrics for the first time. We describe the status quo of articles published in this field since the emergence of research, and provide a comparative analysis of China and the United States.

In this study, 943 articles were included and publication trends were predicted. According to our logistic model, 220 articles are expected to be published in 2028. China and the United States are the 2 countries with the highest number of publications, their cooperation in this field is the closest in the world, and their influence on this field is far-reaching.

Both Chinese and American studies focus on Neurosciences, Neurology, and Cell Biology. However, China’s recent move to the category of Medicine, Research, & Experimental indicates that research is focused on exosomal delivery as a treatment for stroke. Moreover, targeted delivery of exosome miR-124 has been shown to play a key role in neuroprotection during cerebral ischemia.^[15,21]^ The shift in the United States to “Chemistry” and “Materials Science” is actually studying exosomes as drug delivery carriers for treating brain injuries.^[22,23]^

Interestingly, although there is more literature published by China, the intensity and number of citation mutations in discipline direction are not as strong as in the United States, which may reflect the greater proficiency in disciplines in the field in the United States. At the same time, Germany also occupies a place in this field, its cooperation intensity is in the third place, and its authors are more active. The total number of articles published in China, the United States and Germany accounts for most of the research literature (81.68%). This indicates that the research is relatively scattered among other countries, and we speculate that this is because the cooperation intensity of other countries is relatively low and the cooperation is not extensive enough.

According to our analysis, China, Henry Ford Hospital, and Michael Chopp are the country, institution, and author with the highest number of publications in this field. Articles by American author Hongqi Xin not only accounted for 4 of the top 10 co-cited papers but also the top 2 most-cited papers. The author has led the research in this field and was a cornerstone of its emergence.

The research in terms of countries or regions and their cooperation reveals the global attitude and the current situation of research. In general, the cooperation is not close enough. From the regional point of view, Europe is the region with the most cooperation with other continents, as its cooperation intensity is the largest. Asia has the highest number of publications, and its cooperation intensity is obvious. This may be because China has great influence in this field.

Journal analysis provides guidance for choosing suitable journals in which to publish research. In general, the JCR partition and IF of the journals that publish articles about exosomes in stroke are considerable, which indicates that the research in this field is at the forefront. Interestingly, *International Journal of Molecular Sciences* in Q1 was the most active, but its citations per papers count was less prominent than that of other Q1 journals. The possible reason is that it is a relatively new journal as far as publication of articles this field. Relevant articles were last published in *Stroke* in January 2017, which is close to the earliest publication time of this journal. Thus, time difference for the last publication in *Stroke* versus *International Journal of Molecular Sciences* is large. Hence, *Stroke* is more well-known and has an advantage in citation times.

Among the institutions, Henry Ford Hospital is the most active one, probably because the top 2 active authors Michael Chopp and Zheng Gang Zhang, and the important author Hongqi Xin work there. However, the institution is not as cooperative as Oakland University, the third most active university. Southeast university (Nanjing, Jiangsu, China) was in the top 5 cooperative institutions but with relatively few articles. The Essential Science Indicators ranking shows that active institutions are more fragmented, which may be due to the choice of disciplines or not being closely related to the field.

Cluster analysis of keywords can provide a new angle for systematically studying the hotspots.^[24]^ The cluster analysis of keywords included 5 clusters. “Exosomes and the Mechanism of Stroke: Inflammation and Apoptosis” was the largest cluster, which indicates that exosomes are related to the internal mechanism of stroke, and there are many studies. Exosomes, as the communication media in cells, are composed of lipid bilayer membrane structures and carry RNA (mRNA, microRNA, and other noncoding RNA), DNA, protein, glycan, and lipid.^[25]^ The exosomes were found to be related to the mechanism of stroke, which also manifested in many aspects. First of all, exosomes are secreted by many kinds of cells, and many kinds of MSC-derived exosomes have been shown to exhibit functional recovery and vascular repair,^[26]^ which are conducive to alleviating cerebral ischemia–reperfusion injury. Secondly, microRNA in exosomes can play diversified roles, such as reducing neuroinflammation^[27]^ and exerting neuroprotective effects^[28,29]^ (Inflammation was one of the high-frequency keywords in this cluster.) According to the analysis of the keyword timeline, the future research direction may still focus on MSCs.

The mutation of keyword citation times can be used as a key indicator.^[30]^ The keyword of the recent mutation is “circulating microRNA,” which may be used as a diagnostic indicator because the level of some circulating exosomes (such as miRNA-223 and miRNA-17-5p) will increase after cerebral ischemia.^[31,32]^ To some extent, this finding denotes the clinical significance of exosomes in cerebral ischemia.

By analyzing the mutations of keywords between China and the United States and global keywords, it could be seen that many global keyword mutations are caused by China and the United States. For example, “death” broke out in China in 2020 to 2021, whereas in the world, it broke out in 2020 to 2021. “Neurovascular plasticity” broke out in the United States in 2016 to 2019, which corresponded to the world’s outbreak in 2016 to 2018. The keyword “contributes” with the highest mutation intensity in the world was in 2018 to 2020, but the keyword mutation in China occurred in 2017 to 2020. One can speculate that global research on this degenerative disease might have influenced the research in China.

Stem cell therapy is the focus of regenerative medicine research. Neural stem cell therapy was found to promote angiogenesis in rodents with positive effects on cerebral blood perfusion.^[33]^ It seems to hold promise for the limited treatment options available for stroke, but the underlying mechanisms remain elusive.^[34]^

Stem cell transplantation can exert paracrine effects, and exosomes play an important role in paracrine effects.^[35]^ The emergence of exosomes is a new possibility, and because of their low immunogenicity and targeting effect, the use of exosomes has become a new treatment option.^[36]^ In our bibliometric analysis, although “cell therapy” appeared in the keyword and reference clustering, the co-occurrence and time intensity of “extracellular vesicles” were the largest. These findings confirm the leading role of cell therapy in exosome research.

There are many sources of exosomes, however, such as MSCs, tumor cells, and macrophages. In fact, research in this field cannot be done without exosomes secreted by MSCs. The MSC-derived secretome contains a mixture of growth factors that promote tissue repair and regeneration and new angiogenesis. Studies have shown that MSC treatment can still significantly improve ischemic brain injury, independent of cell migration to the ischemic site.^[37]^ In their review, Cunningham et al described the role of the MSC secretome in promoting repair in preclinical models of stroke as occurring through a variety of mechanisms including reduced neuroinflammation, neuroprotection, increased angiogenesis, and neurogenesis.^[38]^ There is also growing interest in cell-free methods, such as exosomes or conditioned medium, which should be studied more comprehensively. The role of exosomes as delivery vehicles in ischemic stroke has been recognized. Researchers have used gold nanoparticles as markers for exosome neuroimaging in vivo, demonstrating the superiority of exosomes in treatment of various brain pathologies (including stroke, Alzheimer disease) and targeted drug delivery applications.^[39]^

From cell therapy to exosome therapy, a cell-free therapy, various research directions have been put forward. Exosomes mediate microRNA to promote the growth and repair of nerve cells.^[21,40]^ In addition, the idea of using exosomes as clinical carriers for drug delivery has been widely recognized.^[40,41]^ Clinically, the role of exosomes in stroke has been confirmed, but the mechanism is still under further study.

It can be seen from our bibliometric and visual analyses that the study of exosomes in stroke is necessary but still needs more in-depth investigation. Additional clinical trials are needed for confirmation. However, many researchers are optimistic about exosomes as a cell-free therapy, and the overall trend of exosome research is good.

## 5. Chinese medicine and exosomes

Exosomes, derived from various cell types with a size range from similar to 40 to 160 nm in diameter, have gained attention recently. Natural compounds are known to be one of the main constituents of traditional Chinese medicine (TCM).^[42]^ Exosomes can also be considered to ameliorate natural compounds, the main constituents of TCM, which are usually ignored due to the complexity of their structures, poor stability, and unclear mechanisms of action.^[43]^

At the same time, Chinese medicine can alleviate the disease by treating exosomes. Shikonin, as a naphthoquinone isolated from the traditional Chinese medicine Lithospermum, can inhibit the proliferation of MCF-7 cells through reducing tumor-derived exosomal miR-128. And it suggests that shikonin suppresses MCF-7 growth by the inhibition of exosome release.^[44]^ Danhong Injection and Danhong Injection-induced exosomes inhibited apoptosis, promoted the miR-125b expression level, and regulated the p53 apoptotic pathway in postinfarction myocardium.^[45]^

As for ischemic stroke, research shows that Buyang Huanwu Decoction, a classic TCM decoction, is promising to alleviate neurological impairment after ischemic stroke. The mechanism is extracellular vesicles from medicated plasma of Buyang Huanwu decoction-preconditioned neural stem cells accelerate neurological recovery following ischemic stroke.^[46]^

## 6. Chinese medicine and stroke

### 6.1. Gualou Guizhi decoction

Gualou Guizhi decoction (GLGZD), a well-established traditional Chinese medicinal formulation, is composed of 6 herbs, including *Ramulus cinnamomi*, *Trichosanthis radix*, *Glycyrrhiza*, *Paeonia lactiflora*, *Zingiber officinale* Roscoe, and *Fructus jujubae*, based on traditional Chinese medical theories “yin yang.” Brain cerebral infarct size was also significantly decreased at day 7 following cerebral ischemia after treated with GLGZD. And GLGZD had a neuroprotective effect in ischemic brain injury, which was due to the inhibition of the inflammatory response and of NF-κB (p65) activation.^[47]^ Studies have shown that GLGZD enhances the absorption and utilization of glucose by nerve cells after CI/R and exerts a significant anti-inflammatory effect by regulating the polarization of microglia.^[48]^ The findings of another study demonstrated that GLGZD facilitated neural function recovery through the promotion of cell proliferation, enhancement of axon regeneration, and augmentation in the population of neuronal precursors and astrocytes within the peri-infarction region.^[49]^ In conclusion, GLGZD can alleviate the damage caused by cerebral ischemia and produce anti-inflammatory and repair effects.

### 6.2. Huanglian-Jie-Du Decoction

Huanglian-Jie-Du Decoction (HLJDD), also known as Oren-gedoku-to in Japanese, is an important and classical multi-herb remedy in TCM. HLJDD is composed of *Rhizoma coptidis*, *Radix scutellariae*, *Cortex phellodendri*, and *Fructus gardeniae* at a ratio of 3:2:2:3. Studies have shown that active ingredients derived from HLJDD could protect against cerebral ischemia via anti-apoptosis effects. Baicalin can obviously reduce the nerve function defect and reduce the volume of cerebral infarction, and the neuroprotective effect of it partially due to myocardin-related transcription factor-A-mediated transactivity, which might be controlled by the activation of phosphatidylinositol-3kinase and extracellular signal regulated kinase-1/2.^[50]^

### 6.3. Bu-yang-huan-wu-tang

Bu-yang-huan-wu-tang is a popular traditional Chinese medicine formula consisting of 7 herbal medicines (*Astragalus membranaceus*, *Angelica sinensis*, *Paeonia lactiflora*, *Ligusticum chuanxiong*, *Carthamus tinctorius*, *Amygdalus persica*, and *Pheretima aspergillum*), that has been the most common prescribed Chinese herbal formula for stroke patients.^[51,52]^ The mechanism, however, is still being investigated.

## 7. Strengths and limitations

This study uses a scientific metrology analysis method to provide a more comprehensive and intuitive overview of the knowledge framework and research status of exosomes and stroke from 2008 to 2023.The study, however, is not without its limitations and deficiencies. Firstly, although the WoSCC database is the most commonly used database in bibliometrics, our study only focused on SCI-Expanded in the WoSCC database, which may exclude studies of related databases such as pubmed.^[53–58]^ Secondly, we did not include data from papers published in 2024 in our bibliometric analysis because the database is regularly updated and the dataset for this year is not yet complete. Finally, some recently published high-quality studies may have been underestimated in our analysis due to limited frequency of citations.

## 8. Conclusion

Our study conducted bibliometric and visual analyses of the published articles on exosomes in stroke. These articles have been published mainly by China and the United States. Since 2013, the number of publications has increased year-by-year, indicating that this field has gained more attention. However, by 2021, the rate of growth in the field has slowed down, possibly because related research has reached a mature stage. Otherwise, overall international cooperation and institutional cooperation in this field need to be strengthened. There are similarities and differences in research between China and the United States, and these 2 countries cooperate closely. The recent research focus is on “circulating microRNA,” whereas the research focus of China and the United States with the most published articles is “stem cells.” Our study provides insights into the current status of stroke and exosome research, which may indicate its future trends. According to our keyword timeline analysis, the future research direction may still focus on exosomes derived from MSCs. Our study findings may enable researchers to select appropriate collaborative institutions, collaborators and journals for publication, as well as promote the in-depth study of exosomes in stroke.

## Author contributions

**Data curation:** Kaihan Zhan.

**Investigation:** Jiayu Li.

**Methodology:** Jiayu Li.

**Project administration:** Li Yu.

**Resources:** Saiqi Cheng.

**Supervision:** Xiaohong Li.

**Visualization:** Jiaqi Song.

**Writing – original draft:** Jiaqi Song.

**Writing – review & editing:** Jiaqi Song.

## Supplementary Material


