# Supplementary material for: Bibliometric and visual analyses of research on the links between stroke and exosomes from 2008 to 2023
Source: Medicine (Baltimore). 2024 Sep 6;103(36):e39498. doi: 10.1097/MD.0000000000039498 (PMC11384054; doi:10.1097/MD.0000000000039498)
Supplement: Supplementary file 1 [file medi-103-e39498-s001.docx]

Supplementary Material

# Supplementary Figures and Tables

Table 1 The top 5 organizations with total connection strength.

| Rank | Organization | Total link strength |
| --- | --- | --- |
| 1 | Oakland university | 39873 |
| 2 | Henry ford hospital | 35990 |
| 3 | Shanghai jiao tong university | 29722 |
| 4 | University duisburg essen | 22135 |
| 5 | Southeast university | 17829 |

Supplementary Material

Table 2 The top 10 keywords in terms of frequency.

| Rank | Keywords |
| --- | --- |
| 1 | extracellular vesicles |
| 2 | exosomes |
| 3 | stroke |
| 4 | functional recovery |
| 5 | ischemic stroke |
| 6 | brain |
| 7 | inflammation |
| 8 | stromal cells |
| 9 | exosome |
| 10 | injury |

Supplementary Material

Table 3 The label of the reference co-cited map.

| Clustering of clusters | Title of label |
| --- | --- |
| #0 | **ischemic stroke; cell-derived exosome; mesenchymal stem; central nervous system diseases; exosomal mirna \| extracellular vesicle; exosomal microrna; stroke treatment; neuroprotective effect; stromal cell** |
| #1 | extracellular vesicle; ischemic stroke; cell-derived extracellular vesicle; mesenchymal stem; cell-derived exosome \| stem cell; stroke treatment; potential therapeutic mechanism; porcine model; therapeutic potential |
| #2 | ischemic stroke; mesenchymal stem; functional recovery; cell-derived exosome; neural stem \| extracellular vesicle; emerging prospect; brain disease theranostics; transcriptional change; injuries-are extracellular vesicle |
| #3 | extracellular vesicle; ischemic stroke; central nervous system diseases; recent advance; brain disease theranostics \| cell-derived exosome; current perspective; brain diseases; cerebral ischemia-reperfusion injury; neurological disorder |
| #4 | stroke pathogenesis; non-coding rna; neural stem cell; lschemic brain; molecular diagnostics \| cardiovascular disease; hypoxia-associated tumour development; extracellular vesicle; stroke prevention; prognostic potential |
| #5 | translation arrest and ribonomics in post-ischemic brain: layers and layers of players |
| #6 | multipotent mesenchymal stromal cell; exosome-mediated transfer; neural cell; functional recovery; mediating cell-based therapy \| role; stroke; mediating exogenous cell-based restorative therapy; astrocyte; neural plasticity |
| #7 | ischemic stroke; extracellular vesicle; mesenchymal stem; mesenchymal stem cell; ischemic stroke treatment \| stroke treatment; exosome therapy; 19-induced stroke; using mesenchymal stem; cells-derived extracellular vesicle |
| #8 | ischemic stroke; central nervous system diseases; extracellular vesicle; therapeutic effect; neurodegenerative diseases \| recent advance; ischemic stroke treatment; new brain-targeting strategies; tissue repair; inflammatory regulation |
| #9 | endothelial cell; endothelial progenitor cell; cns diseases; new diagnostic tool; novel method \| nanoparticle tracking analysis; using microbead; vascular biology; working group; diseases position paper |
| #10 | circulating micrornas: association with disease and potential use as biomarkers |
| #11 | microparticles in physiological and in pathological conditions |
| #12 | extracellular vesicle; mesenchymal stem; neuroprotective effect; cell-derived extracellular vesicle; stem cell secretome \| ischemic stroke; potential target; astrocyte extracellular vesicle; small extracellular vesicle; tissue damage |
| #13 | epileptiform activity induces vascular remodeling and zonula occludens 1 downregulation in organotypic hippocampal cultures: role of vegf signaling pathways |
| #14 | diagnostic tools for hypertension and salt sensitivity testing |
| #15 | the role of astrocytes in mediating exogenous cell-based restorative therapy for stroke |
| #16 | extracellular vesicle; ischemic stroke; cell-derived extracellular vesicle; mesenchymal stem; cerebrovascular diseases \| therapeutic potential; functional recovery; novel approach; cns drug delivery tool; comprehensive analysis |
| #18 | neural stem cell transplantation therapy; ischemic stroke; potential therapeutic role; ischemia-reperfusion injury; stem cell \| ischemia-reperfusion injury; potential therapeutic role; stem cell; neural stem cell transplantation therapy; stroke-associated pneumonia |
| #20 | extracellular vesicle; ischemic stroke; neurovascular unit; blood-brain barrier; cellular atp level \| potential biomarker; ameliorate neuronal damage; astrocyte-derived exosome; neuronal extracellular vesicle; salvageable neuron |

Supplementary Material

Table 4 The label of the keyword co-citation map.

| Clustering of clusters | Title of label |
| --- | --- |
| #0 | **extracellular vesicle; ischemic stroke; atrial fibrillation; acute ischemic stroke; endothelial cell \| mesenchymal stem; cell-derived exosome; cell-derived extracellular vesicle; functional recovery; mesenchymal stem cell** |
| #1 | extracellular vesicle; ischemic stroke; stem cell; mesenchymal stem cell; mesenchymal stem \| cell-derived exosome; central nervous system diseases; bone marrow; ischemic stroke treatment; cns diseases |
| #2 | ischemic stroke; mesenchymal stem cell; extracellular vesicle; mesenchymal stem; functional recovery \| neurological function; acute ischemic stroke model; cerebral endothelial cell-derived small extracellular vesicle; embolic stroke treatment; neurovascular function |
| #3 | extracellular vesicle; neurological disorder; cns diseases; blood-brain barrier; novel biomarker \| mesenchymal stem; mesenchymal stem cell; cell-derived exosome; cardiovascular disease; central nervous system diseases |
| #4 | ischemic stroke; mesenchymal stem cell; mesenchymal stem; stem cell; cell-derived exosome \| extracellular vesicle; traumatic brain injury; cell-derived extracellular vesicle; neurological disorder; extracellular vesicle-based therapeutics |
| #5 | extracellular vesicle; ischemic stroke; mesenchymal stem; ischemic brain injury; neuroprotective effect \| m2 microglia; bone marrow; mesenchymal stem cell; non-coding rna; secretory protein |
| #6 | extracellular vesicle; cerebrovascular diseases; cardiovascular diseases; cerebral ischemia; nitinol material \| ischemic stroke; acute ischemic stroke; promising therapeutic target; affinity enrichment; mrna change |
| #7 | extracellular vesicle; targeted delivery; reperfusion injury; cell-derived extracellular vesicle; traumatic brain injury \| high mobility group; using brain-targeting exosome; box-1 sirna; brain disease theranostics; cns drug delivery tool |
| #8 | pathological condition; 126-modified adsc; suppressing microglia activation; functional recovery; extracellular vesicle \| clinical trial; molecular mechanism; venous thromboembolism; recent advance; pathological condition |
| #9 | mitochondrial dna (mtdna) a3243g mutation associated with an annular perimacular retinal atrophy |
| #10 | nanotheranostics of circulating tumor cells, infections and other pathological features in vivo |
| #11 | central role of mitochondrial injury in the pathogenesis of acute pancreatitis |
| #12 | diagnostic tools for hypertension and salt sensitivity testing |
